# miR-381-3p suppresses breast cancer progression by inhibition of epithelial–mesenchymal transition

**DOI:** 10.1186/s12957-021-02344-w

**Published:** 2021-08-06

**Authors:** Yong-Zheng Yu, Qiang Mu, Qian Ren, Li-Juan Xie, Qi-Tang Wang, Cui-Ping Wang

**Affiliations:** 1 The First Department of Breast Surgery, Affiliated Qingdao Central Hospital, Qingdao University, Qingdao, 266042 China; 2 Department of Ophthalmology, Qingdao Women and Children’s Hospital, Qingdao University, Qingdao, 266034 China

**Keywords:** miR-381-3p, Epithelial, Mesenchymal transition, Transforming growth factor-β, Progression, Breast cancer

## Abstract

**Background:**

Accumulating evidence indicates that miRNAs are involved in multiple cellular functions and participate in various cancer development and progression, including breast cancer.

**Methods:**

We aimed to investigate the role of miR-381-3p in breast cancer. The expression level of miR-381-3p and EMT transcription factors was examined by quantitative real-time PCR (qRT-PCR). The effects of miR-381-3p on breast cancer proliferation and invasion were determined by Cell Counting Kit-8 (CCK-8), colony formation, and transwell assays. The regulation of miR-381-3p on its targets was determined by dual-luciferase analysis, qRT-PCR, and western blot.

**Results:**

We found that the expression of miR-381-3p was significantly decreased in breast cancer tissues and cell lines. Overexpression of miR-381-3p inhibited breast cancer proliferation and invasion, whereas knockdown of miR-381-3p promoted cell proliferation and invasion in MDA-MB-231 and SKBR3 cells. Mechanistically, overexpression of miR-381-3p inhibited breast cancer epithelial–mesenchymal transition (EMT). Both Sox4 and Twist1 were confirmed as targets of miR-381-3p. Moreover, transforming growth factor-β (TGF-β) could reverse the effects of miR-381-3p on breast cancer progression.

**Conclusions:**

Our observation suggests that miR-381-3p inhibits breast cancer progression and EMT by regulating the TGF-β signaling via targeting Sox4 and Twist1.

## Introduction

Breast cancer is one of the most common malignant tumors and is the second leading cause of death from cancer in females [1]. Although the advances in diagnosis and systemic therapy have considerably improved the survival of patients with breast cancer, the survival of patients with metastatic breast cancer remains only 12–24 months following the diagnosis of metastasis [2]. Therefore, it is of great importance to investigate the underlying mechanism of breast cancer development and progression, and identification of new therapeutic target molecules, which will possess important theoretical and practical significances for the development of novel therapeutic strategies.

MicroRNAs (miRNAs) are endogenous small non-coding RNA molecules containing about 22 nucleotides and regulate the expression of target genes at the post-transcriptional level by binding to the 3′-untranslated regions (3′-UTRs) of the target mRNA, resulting in gene silencing by translational repression or mRNA degradation [3]. Accumulating evidence indicates that miRNAs are involved in multiple cellular functions and participate in a number of physiological and pathological processes, including differentiation, cell proliferation, senescence, metabolism, and apoptosis [4, 5]. The abnormal expression of miRNAs is observed in most all of human cancers during cancer development and progression, including breast cancer [6]. miR-381 has been reported to function as a tumor suppressor in various types of cancers, including pancreatic cancer [7, 8], colorectal cancer [9–11], hepatocellular carcinoma [12], lung cancer [13–16], prostate cancer [17, 18], gastric cancer [19, 20], cervical cancer [21, 22], bladder cancer [23], osteosarcoma [24], and breast cancer [25–27]. Although several studies have been demonstrated that miR-381 could be functioned as a tumor suppressor in breast cancer, the functional involvement and the target genes of miR-381-3p are still limited.

Epithelial–mesenchymal transition (EMT) is a cellular process that epithelial cells acquired the mesenchymal phenotype during tissue fibrosis, embryonic development, and cancer progression [28, 29]. During the EMT process, epithelial cells lose cell–cell adhesion and acquire a fibroblastoid morphotype with invasive and migratory properties and EMT contributes to cancer progression, metastasis, and therapeutic resistance [30]. Thus, targeting the EMT signaling appears to be a promising strategy in cancer therapy. Numerous studies showed that miRNAs participate in EMT during breast cancer progression [31].

In the present study, we aimed to investigate the role and underlying molecular mechanism of miR-381-3p in breast cancer progression. We also revealed that the EMT-related transcriptional factors, Twist1 and Sox4, are the potential targets of miR-381-3p. miR-381-3p regulates the breast cancer progression and EMT through the transforming growth factor-β (TGF-β) signaling.

## Materials and methods

### Human specimens

The primary breast cancer and the paired adjacent normal breast tissues were collected from surgical specimens from 20 patients with breast cancer at Qingdao Central Hospital. After radical prostatectomy, the breast cancer tissues and the paired adjacent normal tissues were flash-frozen in liquid nitrogen and stored at − 80 °C. All the patients signed informed consent forms before surgery. This study was approved by the ethics committee of the Qingdao Central Hospital.

### Cell lines and transfection

Human epithelial cell line MCF10A and breast cancer cell lines MCF7, T47D, BT549, MDA-MB-231, and SKBR3 were obtained from the Type Culture Collection of the Chinese Academy of Sciences (Shanghai, China). MCF10A cells were cultured in DMEM/F12 (Hyclone, Logan, UT) supplemented with 5% horse serum (Life Technologies, Grand Island, NY), 20 ng/ml epidermal growth factor (R&D Systems, Minneapolis, MN), 10 μg/ml insulin (Sigma-Aldrich, Milwaukee, WI), 0.5 μg/ml hydrocortisone (Sigma-Aldrich), and 0.1 μg/ml cholera toxin (Sigma-Aldrich). BT549, MDA-MB-231, and SKBR3 cells were maintained in RPMI-1640 (Hyclone) supplemented with 10% fetal bovine serum (FBS, Life Technologies). MCF7 and T47D cells were cultured in DMEM (Hyclone) with 10% FBS. All cells were supplemented with 100 mg/ml streptomycin (Hyclone) and 100 IU/ml penicillin (Hyclone) and cultured in 5% CO_2_ atmosphere at 37 °C.

miR-381-3p mimics, inhibitors, and negative control oligonucleotides were purchased from RiboBio (Guangzhou, China). Transient transfection was carried out using Lipofectamine 3000 (Thermo Fisher Scientific, Waltham, MA) according to the manufacturer’s recommendation.

### Quantitative real-time PCR (qRT-PCR)

Total RNA was isolated from the human frozen tissues or cells using TRIzol reagent (Thermo Fisher Scientific) according to the manufacturer’s instructions. RNA was reverse transcribed into cDNA using a PrimeScript RT reagent kit (Takara, Dalian, China). qPCR analysis was performed on CFX96 Touch™ (Bio-Rad, Hercules, CA) using SYBR Green Mix (Takara). U6 and GAPDH housekeeping genes were used for the internal controls. Data were analyzed using the 2^−ΔCT^ method. The qPCR primers are listed in Table 1.

**Table 1:** Primers used in qRT-PCR

Name	Sequence
miR-381-3p	5′-TCAGACGACAACCGTCTGTG-3′
	5′-AAAATTGAGCACCAACGGGC-3′
miR-200	5′-ACACTCCAGCTGGGTAACACTGTCTGGTAACG-3′
	5′-CTCAACTGGTGTGGTGGAGTCGGCAATTGAGTTGAGACATCGTT-3′
U6	5′-CTCGCTTCGGCAGCACA-3′
	5′-AACGCTTCACGAATTTGCGT-3′
Slug	5′-ATACCACAACCAGAGATCCTCA-3′
	5′-GACTCACTCGCCCCAAAGATG-3′
Snail	5′-GCAAATACTGCAACAAGG-3′
	5′-GCACTGGTACTTCTTGACA-3′
Sox4	5′-CTGCGCCTCAAGCACATG-3′
	5′-TTCTTCCTGGGCCGGTACT-3′
Twist1	5′-GGAGTCCGCAGTCTTACGAG-3′
	5′-TCTGGAGGACCTGGTAGAGG-3′
Zeb1	5′-TGCACTGAGTGTGGAAAAGC-3′
	5′-TGGTGATGCTGAAAGAGACG-3′
Zeb2	5′-CGCTTGACATCACTGAAGGA-3′
	5′-CTTGCCACACTCTGTGCATT-3′
Tgfb1	5′-CCAACTATTGCTTCAGCTCCA-3′
	5′-TTATGCTGGTTGTACAGGG-3′
Tgfb2	5′-CTGATCCTGCATCTGGTCACG-3′
	5′-TGGGGGACTGGTGAGCTTC-3′
GAPDH	5′-ATGACCCCTTCATTGACCTCA-3′
	5′-GAGATGATGACCCTTTTGGCT-3′

### Western blot

Total protein was extracted from cultured cells using RIPA lysis buffer (Cell Signaling Technology, Danvers, MA) containing PMSF (Cell Signaling Technology). Protein lysates were resolved by SDS-PAGE, transferred to PVDF membranes (Millipore, Bedford, MA), detected with primary antibody overnight at 4 °C, and then incubated with HRP-conjugated secondary antibodies. Western blots were visualized with ECL reagent (Millipore). Antibodies against N-cadherin, E-cadherin, Vimentin, SOX4, Twist1, ER, PR, HER2 (Santa Cruz, Santa Cruz, CA), Smad2/3, p-Smad2, and GAPDH (Cell Signaling Technology) were used.

### Luciferase reporter assay

Wild or miR-381-3p binding site-mutated psiCHEK2-Sox4 or psiCHEK2-Twist1 luciferase reporter plasmid was transfected into MDA-MB-231 cells with miR-381-3p mimics or negative control. After 48 h, the cell lysates were measured for luciferase activity according to the manufacturer’s instructions (Promega, Madison, WI). The firefly luciferase activity was normalized to *Renilla* luciferase activity.

### Colony formation assay

Breast cancer cells were seeded in a 6-well plate at 500 per well. For transient transfection with miR-381-3p mimics, miR-381-3p inhibitor, or negative control, the cells were cultured for 20 days at 37 °C, colonies were washed with PBS, fixed, and stained with hematoxylin. The colonies with more than 50 cells were counted under a microscope.

### Cell viability assay

Cell Counting Kit-8 (CCK-8, Dojindo, Japan) was performed to determine cell viability according to the manufacturer’s instructions. Briefly, cells were seeded in a 96-well plate at 5 × 10^3^ per well. After transfection for 24 h, 10 µl CCK-8 reagent was added to each well and incubated at 37 °C for 2 h before each harvest time. The absorbance of each sample was measured using a microplate reader (Thermo Fisher Scientific) at 450 nm.

### Invasion assay

Cell invasion was assessed by using BD BioCoat Matrigel invasion chambers (BD Biosciences, San Jose, CA). Briefly, the transfected cells were seeded into RPMI-1640 without serum in the upper chamber. The RPMI-1640 with 10% FBS was added to the bottom chamber. After 12 h of incubation, the invaded cells on the lower surface were fixed and stained with crystal violet for 15 min. The invaded cells were photographed under a microscope.

### Statistical analysis

Statistical analyses were performed with the SPSS 20.0 statistical software package (SPSS Inc. Chicago, IL). Data are expressed as the mean ± SD from at least three independent experiments. The Student’s *t*-test (two groups) or ANOVA test (three or more groups) was used to determine the differences between the experimental and control groups. Paired *t*-test was used to determine the difference between breast cancer tissues and normal breast tissues. *P* values less than 0.05 were considered statistically significant.

## Results

### miR-381-3p is down-regulated in breast cancer

**Fig. 1:**
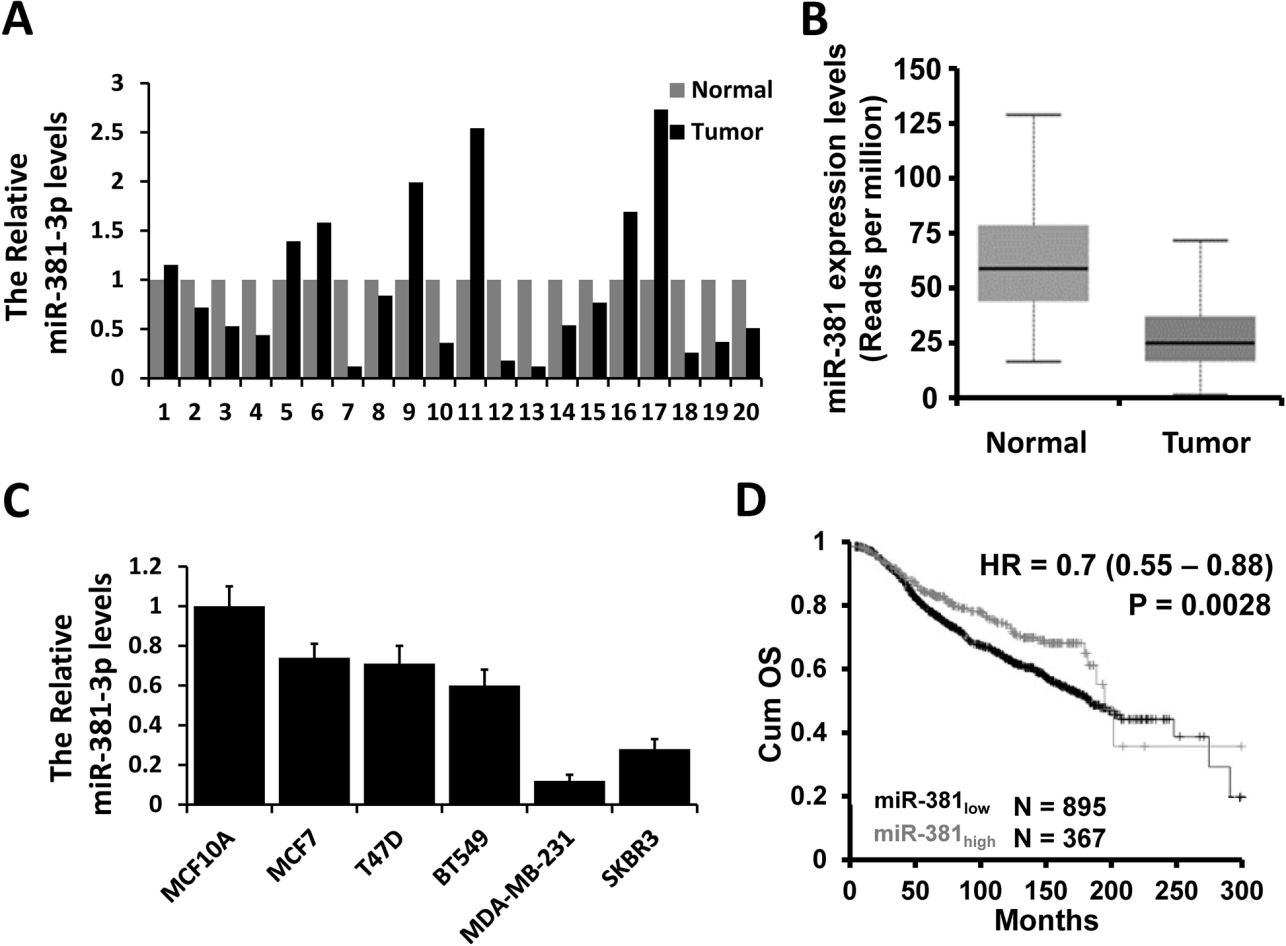
miR-381-3p is down-regulated in breast cancer. **A** The expression level of miR-381-3p in breast cancer and the matched normal tissue samples was examined by qRT-PCR. **B** The expression level of miR-381-3p in breast cancer and the normal tissue samples analyzed in the TCGA database. **C** The expression level of miR-381-3p in breast cancer cell lines and normal cell lines was examined by qRT-PCR. **D** The overall survival was evaluated by KM-Plotter between high and low miR-381-3p expression groups

To determine the role of miR-381-3p in breast cancer, we firstly examined the expression of miR-381-3p in 20 cases of breast cancer tissues and the matched normal breast tissues by qRT-PCR. The expression level of miR-381-3p was significantly decreased in the breast cancer tissue sample (13/20) compared with the matched normal tissue samples (Fig. 1A). This observation was further confirmed in The Cancer Genome Atlas (TCGA) database (Fig. 1B). We next examined the expression of miR-381-3p in five breast cancer cell lines (MCF7, T47D, BT549, MDA-MB-231, and SKBR3) and breast epithelial cell line MCF10A. As shown in Fig. 1C, the breast cancer cell lines had low levels of mIR-381-3p expression compared to MCF10A cells. Furthermore, miR-381-3p down-regulation correlated with poor prognosis in patients with breast cancer by the KM-Plotter database (Fig. 1D). Together, these results suggest that miR-381-3p is decreased in breast cancer and associated with prognosis.

### miR-381-3p inhibits breast cancer progression

**Fig. 2:**
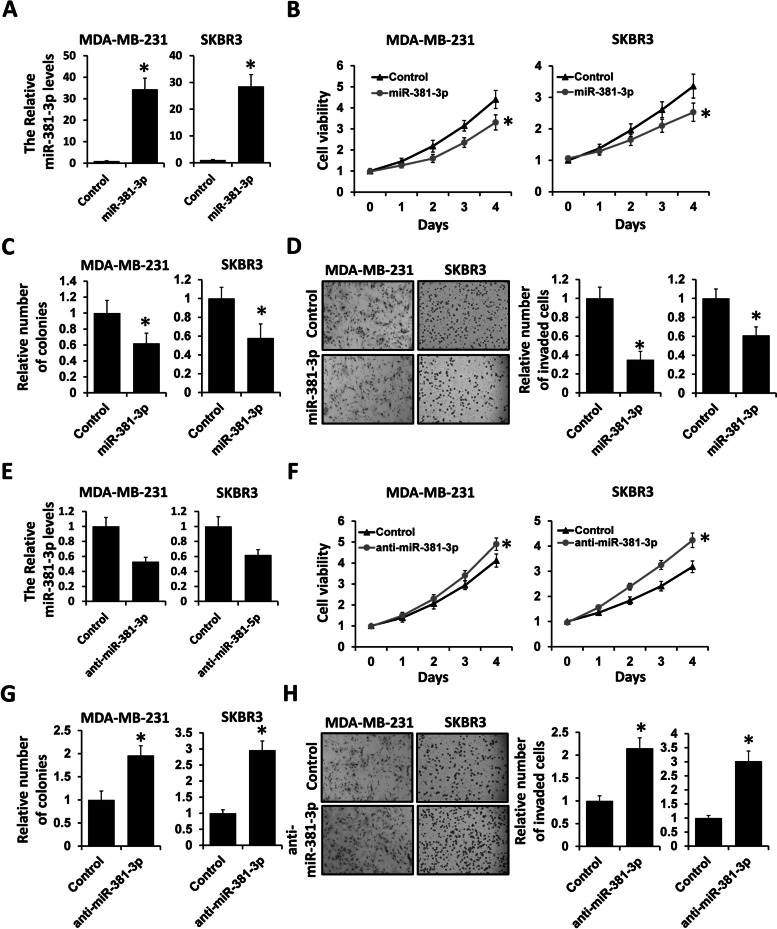
miR-381-3p inhibits breast cancer cell proliferation and invasion. **A** The expression level of miR-381-3p in MDA-MB-231 and SKBR3 cells transfected with miR-381-3p mimics or negative control was examined by qRT-PCR. **B** Cell viability in MDA-MB-231 and SKBR3 cells transfected with miR-381-3p mimics or negative control was assessed by CCK-8 assay. **C** Colony formation analysis of MDA-MB-231 and SKBR3 cells transfected with miR-381-3p mimics or negative control. **D** Cell invasion in MDA-MB-231 and SKBR3 cells transfected with miR-381-3p mimics or negative control was assessed by transwell analysis. **E** The expression level of miR-381-3p in MDA-MB-231 and SKBR3 cells transfected with miR-381-3p inhibitor or negative control was examined by qRT-PCR. **F** Cell viability in MDA-MB-231 and SKBR3 cells transfected with miR-381-3p inhibitor or negative control was assessed by CCK-8 assay. **G** Colony formation analysis of MDA-MB-231 and SKBR3 cells transfected with miR-381-3p inhibitor or negative control. **H** Cell invasion in MDA-MB-231 and SKBR3 cells transfected with miR-381-3p inhibitor or negative control was assessed by transwell analysis. *P < 0.05

To explore the potential role of miR-381-3p in breast cancer progression, we transfected miR-381-3p mimics, miR-381-3p inhibitor, or negative control into MDA-MB-231 and SKBR3 cells. The expression level of miR-381-3p was effectively elevated in MDA-MB-231 and SKBR3 after being transfected with miR-381-3p mimics by qRT-PCR (Fig. 2A). Overexpression of miR-381-3p significantly decreased cell viability in MDA-MB-231 and SKBR3 cells by CCK-8 (Fig. 2B) and colony formation (Fig. 2C) assays. We next assessed the effect of miR-381-3p on breast cancer cell invasion by transwell analysis. As shown in Fig. 2D, the number of invaded cells was significantly decreased in miR-381-3p-overexpressed MDA-MB-231 and SKBR3 cells compared with that in control cells. Conversely, the abilities of cell proliferation and invasion were increased in MDA-MB-231 and SKBR3 transfected with miR-381-3p inhibitor (Fig. 2E–H). Collectively, these results indicated that miR-381-3p functions as a tumor suppressor in breast cancer progression.

### miR-381-3p inhibits EMT in breast cancer

**Fig. 3:**
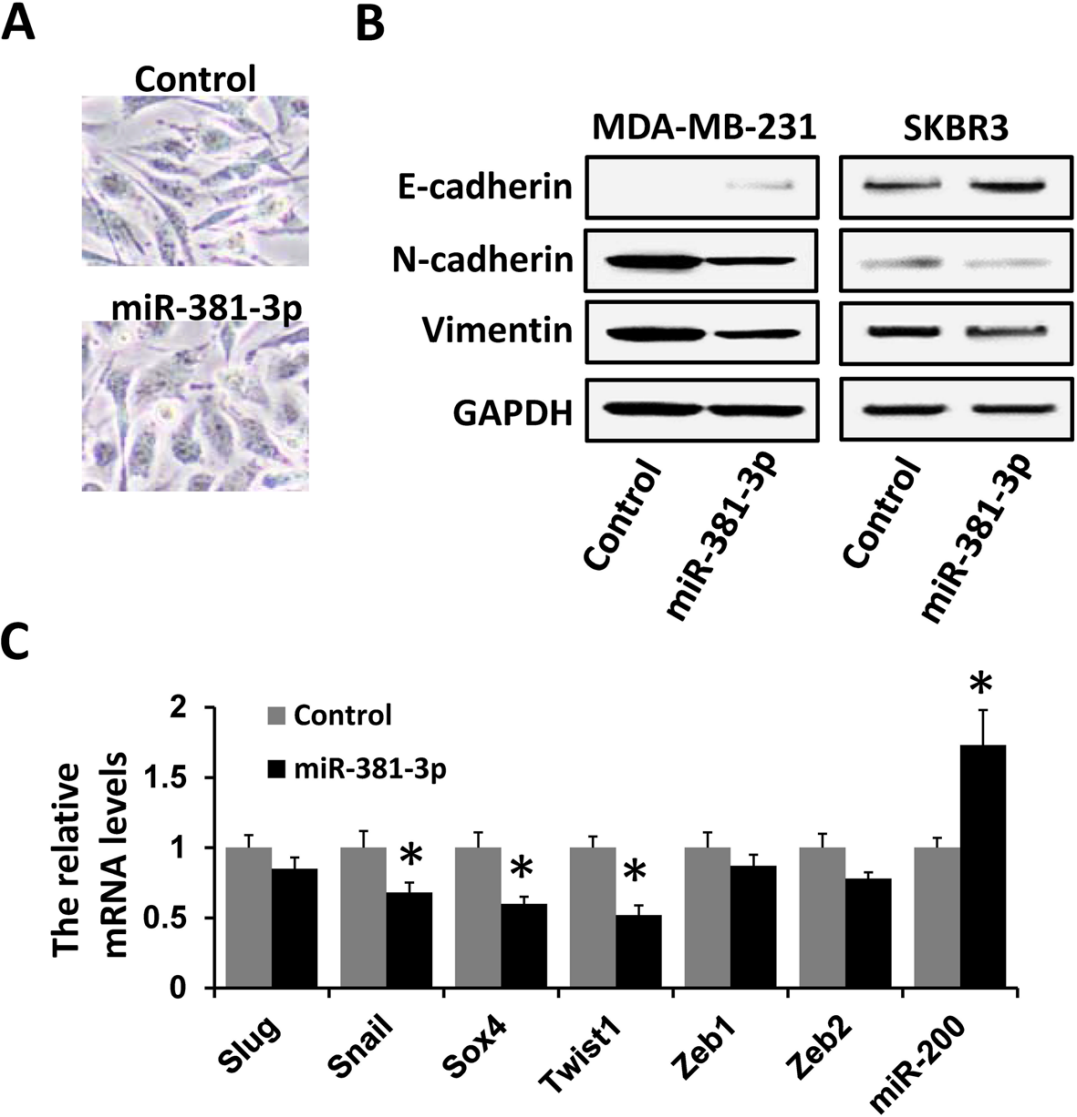
miR-381-5p inhibits EMT in breast cancer. **A** Bright-field images of MDA-MB-231 cells transfected with miR-381-3p mimics or negative control. **B** The expression of E-cadherin, N-cadherin, or Vimentin in MDA-MB-231 cells transfected with miR-381-3p mimics or negative control was examined by western blot. **C** The expression of Slug, Snail, Sox4, Twist1, Zeb1, Zeb2, and miR-200 in MDA-MB-231 cells transfected with miR-381-3p mimics or negative control was examined by qRT-PCR. *P < 0.05

Accumulating evidence suggests that EMT could promote progression in breast cancer. Therefore, we investigated whether miR-381-3p regulates EMT to affect breast cancer progression. As shown in Fig. 3A, we observed that MDA-MB-231 cells transfected with miR-381-3p mimics had a cobblestone-like morphology, whereas control cells maintained their spindle-like fibroblast morphology. Subsequently, we found that the expression of epithelial marker (E-cadherin) was increased in miR-381-3p-overexpressed MDA-MB-231 or SKBR3 cells; meanwhile, the expression of mesenchymal markers (Vimentin and N-cadherin) was decreased in such cells (Fig. 3B). We next examined the effect of miR-381-3p on the main EMT-related factor expression. The expression level of Snail, Sox4, and Twist1 was significantly reduced, whereas the expression of miR-200 was increased in miR-381-3p-overexpressed MDA-MB-231 cells compared to those in control cells (Fig. 3C). Thus, these results showed that miR-381-3p inhibits EMT in breast cancer.

### Sox4 and Twist1 are the targets of miR-381-3p

**Fig. 4:**
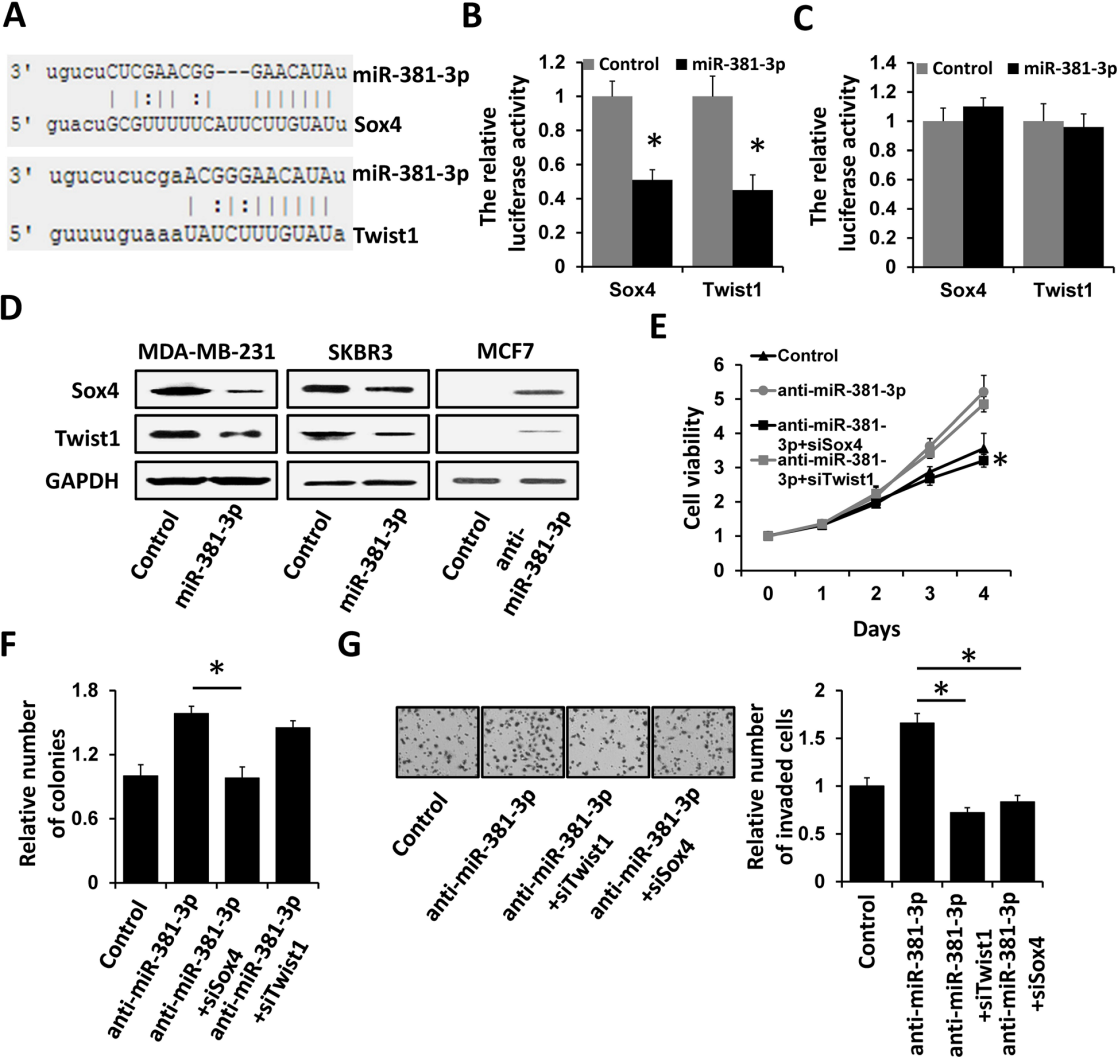
Sox4 and Twist1 are targets of miR-381-3p. **A** Schematic illustration of the predicted miR-381-3p binding sites in Sox4 and Twist1 3′-UTR. **B**, **C** Luciferase reporter assays were performed to demonstrate that miR-381-3p inhibits the wild-type (**B**), but not the mutant (**C**), 3′-UTR of Sox4 or Twist1 reporter activities. **D** The expression of Sox4 and Twist1 in MDA-MB-231 or SKBR3 cells transfected with miR-381-3p mimics or negative control was examined by western blot. **E** Cell viability in T47D cells transfected with miR-381-3p inhibitor or negative control with siRNAs targeting Sox4 or Twist1 was assessed by CCK-8 assay. **F** Colony formation analysis of T47D cells transfected with miR-381-3p inhibitor or negative control with siRNAs targeting Sox4 or Twist1. **G** Cell invasion in T47D cells transfected with miR-381-3p inhibitor or negative control with siRNAs targeting Sox4 or Twist1 was assessed by transwell analysis. *P < 0.05

To investigate whether the EMT-related factors are the targets of miR-381-3p, we analyzed the 3′-UTR of the EMT-related factors by TargetScan. The software predicts that the 3′-UTR of Sox4 and Twist1 harbors a binding site of miR-381-3p (Fig. 4A). We next determined whether miR-381-3p could directly regulate the 3′-UTR of Sox4 and Twist1 by luciferase reporter assay. The 3′-UTRs of Sox4 and Twist1, as well as their mutant containing the putative miR-381-3p binding sites, were cloned into the psiCHEK2 plasmid. These reporter constructs were transfected into MDA-MB-231 cells with miR-381-3p mimics or negative control. The luciferase activity of wild-type Sox4 or Twist1 3′-UTR reporter construct was significantly decreased in the miR-381-3p-transfected MDA-MB-231 compared to those in negative control cells (Fig. 4B). Furthermore, the mutation of miR-381-3p binding sites in these reporter constructs abolished these suppressive effects (Fig. 4C). The expression of Sox4 and Twist1 was significantly reduced in MDA-MB-231 and SKBR3 cells transfected with miR-381-3p mimics compared to those in control cells (Fig. 4D). Moreover, knockdown of Sox4 reversed the effect of miR-381-3p depletion on breast cancer proliferation and invasion in MCF7 cells, whereas knockdown of Twist1 reversed the effect of miR-381-3p depletion on breast cancer invasion, but not altered the proliferation in MCF7 cells (Fig. 4E–G). Thus, these results support the bioinformatics prediction of both Sox4 and Twist1 as direct targets of miR-381-3p.

### miR-381-3p reduces TGF-β signaling-induced breast cancer progression

**Fig. 5:**
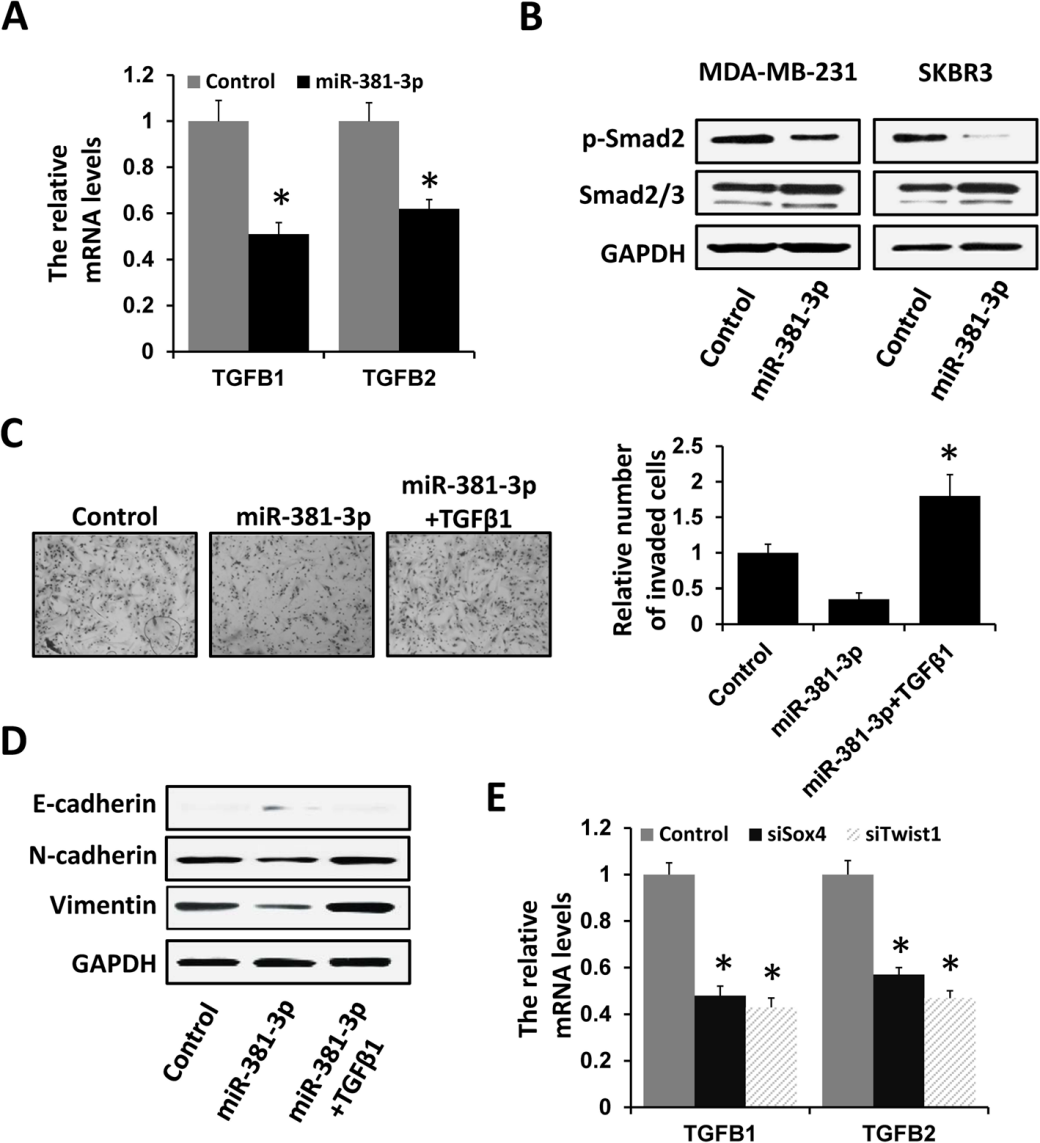
miR-381-3p reduces TGF-β signaling activation in breast cancer. **A** The expression of TGFB1 and TGFB2 in MDA-MB-231 cells transfected with miR-381-3p mimics or negative control was examined by qRT-PCR. **B** The expression of p-Smad2 and Smad2/3 in MDA-MB-231 or SKBR3 cells transfected with miR-381-3p mimics or negative control was examined by western blot. **C** Cell invasion in MDA-MB-231 cells transfected with miR-381-3p mimics or negative control after TGFβ1 addition was assessed by transwell analysis. **D** The expression of E-cadherin, N-cadherin, or Vimentin in MDA-MB-231 cells transfected with miR-381-3p mimics or negative control after TGFβ1 addition was examined by western blot. **E** The expression of TGFB1 and TGFB2 in MDA-MB-231 cells transfected with siRNAs targeting Sox4 or Twist1, as well as a negative control, was examined by qRT-PCR. *P < 0.05

Multiple lines of evidence have implicated that TGF-β signaling is involved in the EMT process in breast cancer progression. We next assessed whether miR-381-3p regulates TGF-β signaling in breast cancer progression. The qRT-PCR analysis revealed a decreased expression of TGFB1 and TGFB2 in miR-381-3p-overexpressed MDA-MB-231 cells (Fig. 5A). In addition, the expression level of p-Smad2, a down-stream effector of TGF-β signaling, was significantly decreased in miR-381-3p-overexpressed MDA-MB-231 cells (Fig. 5B). Moreover, treatment of miR-381-3p-overexpressed MDA-MB-231 cells with TGF-β1 elevated their invasion ability (Fig. 5C) and rescued the expression of EMT biomarker E-cadherin, snail, and vimentin (Fig. 5D). Furthermore, the expression of TGFB1 and TGFB2 was decreased in Sox4- or Twist1-depleted MDA-MB-231 cells (Fig. 5E). These results suggest that miR-381-3p inhibits breast cancer progression through regulating TGF-β signaling by targeting Sox4 and Twist1.

## Discussion

In this study, we demonstrated that miR-381-3p is decreased in breast cancer. miR-381-3p inhibits breast cancer progression and EMT. We identified Sox4 and Twist1 as the direct targets of miR-381-3p. Furthermore, we found that miR-381-3p reduces the TGF-β signaling activation in breast cancer. These results show that miR-381-3p inhibits breast cancer progression through TGF-β signaling by targeting Sox4 and Twist1.

Dysregulation of miRNAs plays an important role in various cancer development and progression, including breast cancer [32, 33]. Aberrant expression of miR-381-3p is observed in many types of cancers, suggesting that miR-381-3p might play a crucial role in tumorigenesis and progression [13, 18, 34–36]. A previous study showed that the expression of miR-381-3p was associated with overall survival in patients with non-small cell lung cancer and miR-381-3p inhibits cancer progression by targeting LRH-1 [37]. miR-381-3p down-regulation was also observed in head–neck squamous cell carcinoma, and miR-381-3p could suppress head–neck squamous cell carcinoma cell progression by targeting nuclear autoantigenic sperm protein [34]. Several studies indicated that miR-381-3p was dysregulated in breast cancer development and progression [27, 38]. miR-381-3p down-regulation contributes to cisplatin or doxorubicin resistance in breast cancer [25, 26, 39, 40]. Consistent with their work, we demonstrated that miR-381-3p is decreased in 13 of 20 patients with breast cancer. The reason is that tissues were acquired by mastectomy, rather than isolating individual normal breast and tumor epithelial cells by microdissection. Moreover, the expression and prognostic value of miR-381-3p in breast cancer should be further confirmed in the future study.

miR-381-3p inhibits breast cancer proliferation and invasion, suggesting that miR-381-3p functions as a tumor suppressor in breast cancer. As the relationship between miRNAs and cancer progression has been gradually understood, miRNA could serve as a prognostic tool in cancer by evaluating the drug sensitivity, predicting recurrence, and estimating the overall survival in patients with cancer [41]. We found that patients with low miR-381-3p expression had a significantly poorer prognosis than those with high miR-381-3p expression by KM-Plotter, suggesting that miR-381-3p down-regulation could serve as a biomarker for poor outcome in patients with breast cancer.

EMT is defined as a process by which epithelial cells lose their cell polarity and cell–cell junction, resulting in changes to cell morphology. EMT acts as a main driver of tumor metastasis, with a crucial role in cancer migration and invasion [28, 42]. Vimentin, N-cadherin, and E-cadherin are generally considered EMT markers. During the EMT process, the expression of Vimentin and N-cadherin (mesenchymal marker) is increased, whereas the expression of E-cadherin (epithelial marker) is decreased. In this study, we observed a decreased E-cadherin expression and an increased Vimentin and N-cadherin expression after transfection with miR-381-3p mimics, suggesting that miR-381-3p inhibits the EMT phenotype in breast cancer. EMT is often driven by changes in gene expression, resulting from the actions of EMT transcription factors. We evaluated the effect of miR-381-3p on the expression of the core EMT transcription factors, including Slug, Snail, Sox4, Twist1, Zeb1, and Zeb2. The expression level of Snail, Sox4, and Twist1 was significantly decreased in miR-381-3p-overexpressed breast cancer cells. Furthermore, miR-381-3p could bind to the 3′-UTR of both Sox4 and Twist1, suggesting that miR-381-3p inhibits breast cancer EMT by targeting Sox4 and Twist1. Despite the evidence of miR-381-3p targeting Sox4 and Twist1, we cannot exclude other key genes that may contribute to the tumor-suppressive role of miR-381-3p.

TGF-β, an established inducer of EMT, is overexpressed in human breast cancer and is correlated with malignant progression and unfavorable outcome in patients with breast cancer [43]. TGF-β induces EMT through a Smad-dependent or Smad-independent pathway. Increasing evidence indicates that miRNAs are involved in TGF-β-induced EMT in breast cancer [44–46]. Our results indicated that miR-381-3p inhibited the TGF-β signaling pathway by dephosphorylation of Smad2. Furthermore, TGF-β1 addition reversed the miR-383-3p-induced suppression of cell invasion in breast cancer. Thus, these results suggest that miR-381-3p inhibits breast cancer EMT by regulation of the TGF-β signaling pathway.

The current study may have many limitations. Firstly, the expression of miR-381-3p was determined in a small number of breast cancer specimens. It is important to validate whether miR-381-3p can be used as a prognostic predictor for patients with breast cancer in a larger specimen study. Secondly, we only assessed the effect of miR-381-3p on breast cancer progression in vitro. Thus, the role of miR-381-3p in breast cancer should be determined in in vivo assays in the future.

## Conclusion

In summary, we showed that miR-381-3p was down-regulated in breast cancer. miR-381-3p inhibits breast cancer progression and EMT by regulating the TGF-β signaling via targeting Sox4 and Twist1.

## Data Availability

All data generated or analyzed during this study are included in this published article and its supplementary information files.
